# 596. Operating Room Burn Skills Day: Building Nursing Knowledge and Confidence

**DOI:** 10.1093/jbcr/irag033.390

**Published:** 2026-04-06

**Authors:** Hailey S Lee, Sheiryl Gica

**Affiliations:** New York Presbyterian Hospital/ Weill Cornell Medical Center, NY, New York; New York Presbyterian Hospital Weill Cornell, Rego Park, NY

## Abstract

**Introduction:**

Operating room nurses are integral part of burn care. In the operating room, they play a key role in preparing supplies, organizing workflows, and supporting the burn surgical team in caring for burn surgical patients. However, many nurses have limited exposure to burn surgeries which can lower their confidence when circulating or scrubbing. We wanted to see if giving staff the opportunity to learn through burn skills day could increase knowledge and in turn, improve their confidence in burn surgeries.

**Methods:**

This is a quality improvement project to improve the operating room nurse' knowledge, and confidence in burn surgeries. We created burn surgery scrub and circulating nurse learning module and conducted a burn skills day. Knowledge and confidence level was assessed after implementation.

**Results:**

Quiz scores showed a significant improvement in nurse's knowledge after burn skills day, increasing by 128%. As knowledge improved, confidence also increased. Confidence survey score increased by 42%. Nurses noted that becoming more familiar with burn supplies, equipment, and workflows made them more comfortable and confident as circulators or scrub nurses.

**Conclusions:**

Burn skills day improved both knowledge and confidence amongst operating room nurses. The combination of teaching, hands-on practice, a burn module gave staff a clear understanding of burn surgery-specific supplies and workflows, which directly increased their confidence when circulating and scrubbing in burn cases. By integrating knowledge with confidence, burn skills day demonstrated its value as an uncomplicated and effective way to prepare the operating room nurses for burn care. Through the positive feedback and support from the surgical team, nursing leadership and nursing staff, a yearly "Burn Skills Day" will be conducted annually.

**Applicability of Research to Practice:**

Applicable to clinical nursing practice.

**Funding for the study:**

N/A.

